# Race, everyday discrimination, and cognitive function in later life

**DOI:** 10.1371/journal.pone.0292617

**Published:** 2023-10-25

**Authors:** Kenneth F. Ferraro, Callie J. Zaborenko

**Affiliations:** 1 Department of Sociology, Purdue University, West Lafayette, Indiana, United States of America; 2 Center on Aging and the Life Course, Purdue University, West Lafayette, Indiana, United States of America; Centre for Demographic Studies, SPAIN

## Abstract

Discrimination is pernicious in many ways, but there are inconsistent findings regarding whether it is harmful to cognitive function in later life. To address the inconsistency, we use two closely related concepts of everyday discrimination to predict cognitive trajectories in a diverse sample. Using data from the Health and Retirement Study (HRS), we examine whether the frequency of discrimination, measured at baseline with six questions, is related to poorer cognitive function and change in function over time (2008–2016). Age at baseline ranged from 53 to 100. Growth curve models of initial cognitive function and change in function were estimated. Everyday *global* discrimination was associated with poorer initial cognition and slower declines over time, and these relationships were not moderated by race and ethnicity. By contrast, the relationship between everyday *racial* discrimination and cognition was moderated by race: more frequent everyday racial discrimination was associated with better initial cognitive function among Black adults but not among Hispanic and White adults. Discrimination is a multifaceted concept, and specific types of discrimination manifest lower or higher cognitive function during later life for White, Black, and Hispanic adults.

## Introduction

After decades of research studying differences in health and cognition by race, the study of discrimination has been a major innovation for identifying how the lived experiences of racial groups are related to health disparities. A substantial literature reveals that discrimination is harmful to physical health [1–3] and mental health [4, 5], and an emerging body of research identifies the toll that discrimination has on cognitive functioning in later life [6, 7].

Despite agreement on the centrality of the concept, debate ensues on the prevalence of discrimination in the U.S. [8–10] and whether it is harmful to cognitive functioning. Although most studies report that discrimination is associated with poorer cognitive function [6, 11, 12], other studies report that discrimination is associated with better cognitive function during later life, at least for persons from selected racial-ethnic populations [13, 14]. There are many plausible reasons for the divergent findings, but two may account for much of the debate. First, findings may differ based on sample composition and study design, especially whether analyses were conducted to examine if relationships differ across groups in diverse samples. Second, there are different types of discrimination, each of which merits study, but it is more challenging to compare findings and develop empirical generalizations derived from distinct but closely related types of discrimination. We address both issues in our review of the literature and analyze whether cognitive functioning in later life is related to two types of discrimination. Unlike many previous studies, we also use longitudinal data to examine trajectories in cognitive functioning.

### Review of empirical evidence

There are different types of discrimination, and most studies using survey data examine everyday discrimination as a chronic stressor—the subjective assessment of being treated unfairly because of membership in a social category [15]. The concept is used mostly to address racial or ethnic discrimination, but there are many additional axes for unfair treatment such as sex, age, use of assistive devices, or bodily appearance (e.g., obesity). Under the rubric of everyday discrimination, there also is a fundamental distinction between unattributed and attributed discrimination [10]. Unattributed everyday discrimination captures the gestalt of discriminatory experiences but is silent on the purported cause of the unfair treatment. By contrast, attributed everyday discrimination identifies one or more causes, typically with a follow-up question for those reporting discrimination [16]. Thus, for the latter type, there are many potential concepts such as everyday racial discrimination, everyday sex discrimination, etc.

Among the studies examining the relationship between discrimination and cognitive function in later life, the majority analyzed everyday discrimination without attribution—what may be referred to as *everyday global discrimination*. Those studies reported that discrimination was associated with poorer cognition [6, 7, 11, 12, 17].

By contrast, a minority used everyday (or daily) discrimination with an attribution to race. Of those examining *everyday racial discrimination*, one study used the information mostly to describe reasons for discrimination mapped onto Black and White respondents of various ages, then analyzed whether unattributed daily discrimination was a mediator of race differences in episodic memory and executive functioning; it was not a mediator [18]. By contrast, another study used the attribution information for race (and eight other reasons) and reached very different conclusions for two cognitive outcomes [14]. The authors reported that everyday racial discrimination was associated with poorer memory at baseline, but it was not related to change in memory over four years. They also reported that “African American participants who perceived race discrimination scored higher” on baseline mental status but not on change over four years (p. 176). Another study reported results for both everyday and major lifetime discrimination on a sample from two California urban areas [13]. Although they did not find that unattributed everyday discrimination was associated with cognitive function, participants who reported race, ancestry, or national origin as the reason for their everyday discrimination manifested higher semantic memory scores. They also reported that major lifetime discrimination attributed to race or ancestry was associated with higher semantic memory. These two studies were the only ones of which we were aware to report *better* cognitive health for Black respondents reporting racial discrimination.

### Theoretical background and hypotheses

Why might discrimination influence cognitive function in later life? Theoretically, discrimination is a stressor that may compromise cognitive function because the act of confronting unfair treatment has cognitive costs—it consumes cognitive resources [19]. Discrimination is a type of social perturbation that requires additional cognitive work, and even subtle forms of discrimination are cognitively taxing [20]. For instance, one recent study of women ages 18–58 showed that subtle discrimination was more consequential than overt discrimination, resulting in poorer task performance [21]. Even though acts of overt discrimination may decrease over time, subtle acts of discrimination may lead to cognitive decline. Regardless of the degree of overtness, if people sense that they have experienced discrimination, it may lead to cognitive decline because of the additional cognitive work required to address unfair treatment.

An alternative and probably less recognized theoretical view is that identifying a specific type of discrimination may aid coping. Although discrimination is often rooted in traumatic experiences [22], identifying a specific reason such as racism for the unfair treatment may help the victim engage with others who were treated unfairly for the same reason. For instance, if one can identify the discrimination as racial, it may lead to “collective coping,” which strengthens identity and exposes the person to alternative ways of handling discrimination [23]. There also may be stressor-specific neuroendocrine responses patterns associated with attributed discrimination [24]. Focusing on resilience in the face of a single or primary reason for discrimination may help people cope with the insult [25]. By contrast, if one identifies multiple reasons for the discrimination arising from various sources, tackling the problem may seem overwhelming.

The thesis of stressor-specific responses also may be consistent with the concept of cognitive reserve whereby people adapt and improvise to reach desired ends and thereby preserve cognitive resources during a period of the life course when cognitive decline is quite common [26]. Stressor exposure challenges the person to respond immediately with cognitive resources that may augment one’s cognitive reserve. In this sense, different types of discrimination could be associated with lower or higher cognitive functioning. Based on the evidence, we specify four main hypotheses, each with two parts addressing (a) initial cognitive function and (b) change in cognition over time.

1. Given that everyday global discrimination is a stressor associated with multiple axes of unfair treatment, we hypothesize that it is associated with poorer cognitive function at (a) baseline and (b) over time.
2. The relationship between everyday global discrimination and cognition varies by race and ethnicity, with Black and Hispanic adults experiencing poorer cognition at (a) baseline and (b) over time.
3. Everyday racial discrimination is associated with poorer cognitive function at (a) baseline and (b) over time.
4. Based on previous research [13, 14], we hypothesize that the relationship between everyday racial discrimination and cognition varies by race and ethnicity, with Black and Hispanic adults experiencing better cognition at (a) baseline and (b) over time.

## Method

### Ethics statement

Panel data from the 2006–2016 waves of the Health and Retirement Study (HRS) were analyzed. Data collection was completed by the University of Michigan’s Survey Research Center. Purdue University’s Human Research Protection Program and Institutional Review Board (IRB) determined that the study involved secondary analysis of data, fully anonymized before the authors accessed the sample, and is therefore exempt (category 4) by U.S. regulations.

### Sample

HRS collects data using a multistage sampling design on individuals 50 years or older, with oversamples of Black adults, Hispanic adults, and Floridians [27]. Response rates for the panel sample exceed 87% for each wave since 2006. The age of respondents in 2006 ranged from 53 to 100.

HRS core interviews occur every two years, and the discrimination questions were introduced in 2006 on a random half-sample. The second half-sample was asked about discrimination in 2008. The data were arrayed so that the discrimination measures reported in 2006 or 2008 were treated as Wave 1 (W1), with cognition measures beginning in 2008 or 2010 (W2), respectively (i.e., 2-year lag between discrimination and cognition). W3 refers to 2010 or 2012 (i.e., 4 years after measuring everyday discrimination, respectively), W4 draws data from 2012 or 2014, and W5 from 2014 or 2016. This data array treats each half-sample equivalently, including the lag structure, but on different schedules.

From a sample of 20,753 cases at W1, we implemented several exclusion criteria for the analysis: (1) had a W1 survey response weight of 0 (resulting in a sample of N = 19,336); (2) did not identify as either White, Black, or Hispanic (N = 18,750); (3) did not provide information on cognition for at least one wave between W2 and W5 (N = 13,154); (4) had dementia or was missing a cognition score at W1 (N = 12,618); and (5) missing a discrimination score at W1 (resulting in N = 11,729). Those excluded differed from the analytic sample in several ways: lower cognition, higher everyday global discrimination and everyday racial discrimination, lower education, younger, lower wealth, lower BMI, less physically active, less multimorbidity, and more depressive symptoms.

### Cognitive function

HRS measures *cognition* using a modification of the Telephone Interview for Cognitive Status that assesses: (1) immediate word recall, (2) delayed word recall, (3) backwards counting, and (4) working memory. The composite measure for the total sample ranges from 0 to 27 and is widely used in studies of cognitive function in later life [28]. For clinical purposes, respondents with a score between 0 and 6 were classified as cognitively impaired due to dementia. Respondents with dementia at 2008–2010 were excluded because they already had the condition, but respondents with dementia onset at subsequent waves were included in the analysis. We examine cognition as a continuous variable, with repeated measurements of cognition for latent growth analysis.

### Everyday discrimination

The HRS measures everyday discrimination based on six questions to assess the frequency of occurrence [15]. Respondents were asked “In your day-to-day life how often have any of the following things happened to you?” The items included (1) treated with less respect, (2) poorer service at restaurants or stores, (3) think you are not smart, (4) afraid of you, (5) threatened or harassed, and (6) poorer service/treatment from doctors or hospitals. Responses were coded from 0 for “never” to 5 for “almost every day.” To create the variable *everyday global discrimination* (EGD), we averaged responses across the six items to reflect the response categories presented to respondents. Respondents with a score of zero reported never experiencing unfair treatment across the six indicators; those scoring five reported almost daily unfair treatment across the six items.

To create the variable *everyday racial discrimination* (ERD), we used the same indicators plus the question about attributions (second stage): “what do you think were the reasons why these experiences happened to you?” Respondents could identify eight reasons (plus “other”) for the discrimination. Examples include race, ancestry or national origin, gender, religion, and age. For this analysis, we created everyday racial discrimination (ERD) when the respondent mentioned race as a reason for unfair treatment, regardless of other reasons selected.

Both EGD and ERD range from 0 to 5, and each respondent has a score on EGD and ERD. (In sensitivity analyses described below, we also re-estimated the models with two additional distinct constructs of ERD to assess robustness of findings across coding strategies).

### Covariates

Models adjust for demographic characteristics, resources, health, lifestyle, and psychological factors in adulthood reported at W1 (2006–2008). Previous studies recommend adjusting for these characteristics because they are related to discrimination [6, 7]. Demographic variables include *age* (in years), *female* (1 = women, 0 = men), and *race and ethnicity* (non-Hispanic White [reference group], non-Hispanic Black, and Hispanic). Resource variables include *education*, measured in years and top coded at 17+, and *wealth*, coded in tens of thousands of dollars and cube rooted to account for skewness. Health variables include *Body Mass Index* (BMI), measured as kilograms divided by meters squared based on self-reported height and weight, and *multimorbidity* is a count of reported chronic diseases that are major causes of death [29, 30]. *Physically active* is a lifestyle variable with binary measurement based on self-reported engagement in moderate or vigorous activities. Psychological variables include *neuroticism*, measured with four items to describe oneself (e.g., moody, nervous), where we averaged responses across the items to reflect the response categories (1, not at all; 4, a lot; α = .71) [31], and *depressive symptoms* (8-item version of CES-D, α = .84) because prior research reveals that persons with more depressive symptoms generally report more everyday discrimination [32]. All covariates were measured in 2006 or 2008, simultaneous with the measurement of everyday discrimination. The range and descriptive statistics for all variables are presented in Table 1.

**Table 1:** Descriptive statistics for total sample and by race and ethnicity, health and retirement study.

Variable (range)	Total (N = 11,729)		White (N = 9,378)		Black (N = 1,426)		Hispanic (N = 915)	
	Mean	SD	Mean	SD	Mean	SD	Mean	SD
Cognition, W1 (7–27) †	15.73	3.84	16.23	3.70	13.68^a^	3.78	13.83^b^	3.69
Cognition, W2 (0–27)	15.27	4.36	15.73	4.18	13.26^a^	4.66	13.60^b^	4.42
Cognition, W3 (0–27)	14.85	4.28	15.35	4.14	12.78^a^	4.24	13.00^b^	4.29
Cognition, W4 (0–27)	14.85	4.53	15.32	4.34	12.85^a^	4.76	13.20^b^	4.77
Cognition, W5 (0–27)	14.55	4.43	15.10	4.25	12.23^a^^c^	4.45	12.72^b^	4.43
EGD (0–5)	0.68	0.78	0.65	0.74	0.86^a^^c^	0.91	0.69	0.90
ERD (0–5)	0.11	0.46	0.03	0.25	0.58^a^^c^	0.88	0.22^b^	0.63
Race and ethnicity††								
White (0,1)	0.80							
Black (0,1)	0.12							
Hispanic (0,1)	0.08							
Age (53–100)	69.00	9.23	69.49	9.29	67.42^a^^c^	8.67	66.49^b^	8.68
Female (0,1)	0.59		0.58		0.66^a^^c^		0.60	
Education (years) (0–17)	12.78	2.94	13.21	2.50	12.11^a^^c^	2.87	9.42^b^	4.51
Wealth (-13.00–34.66)	6.31	3.81	6.91	3.71	3.81^a^	3.12	4.07^b^	3.35
BMI (10.60–68.70)	28.32	5.77	27.93	5.56	30.43^a^^c^	6.65	28.99^b^	5.69
Physically active (0,1)	0.29		0.30		0.22^a^^c^		0.27^b^	
Multimorbidity (0–7)	1.37	1.12	1.34	1.12	1.60^a^^c^	1.14	1.31	1.07
Neuroticism (1–4)	2.35	0.36	2.35	0.35	2.37^a^^c^	0.38	2.22^b^	0.43
Depressive symptoms (0–8)	1.31	1.88	1.19	1.77	1.57^a^^c^	1.99	2.08^b^	2.41

*Notes*: EGD = Everyday global discrimination (unattributed). ERD = Everyday racial discrimination. Wealth is the cube root of thousands of dollars. BMI = body mass index.

^†^ Cognition at baseline was not used in model estimation but to identify and exclude those who had dementia at baseline when EGD and ERD were measured.

^††^ All covariates were measured at baseline.

^a^Significant difference between Black and White adults (p<0.05).

^b^Significant difference between Hispanic and White adults (p<0.05).

^c^Significant difference between Hispanic and Black adults (p<0.05).

### Analytic strategy

The analysis proceeded in three phases, beginning June 1, 2021. First, we examined the distribution of all variables for the total sample and stratified by race and ethnicity. Second, we began by estimating an unconditional latent growth model to examine average trajectories of cognitive function, then sequentially built latent growth models by comparing findings for EGD and ERD on cognition before and after adding the covariates and finally testing for interactions of everyday discrimination by race and ethnicity. Third, we conducted sensitivity analyses, including alternative specifications of ERD. All models were estimated in Stata 15.

## Results

### Descriptive statistics

Descriptive statistics for all study variables are presented in Table 1, both for the total sample and stratified by race and ethnicity. Black and Hispanic older adults report lower levels of cognition than White adults, and values declined slightly in all three groups over time. The two types of discrimination reveal striking differences in the total sample, with EGD at 0.68 and ERD at 0.11. Across the subsamples, EGD is highest among Black adults (0.86), followed by Hispanic (0.69) and White (0.65) adults. For ERD, the racial-ethnic differences are even larger with Black adults reporting the highest value (0.58), followed by Hispanic (0.22) and White adults (0.03). Significant differences exist between Black and White respondents in the means of all covariates. Also, there are significant differences between Hispanic and White respondents in the means for all covariates except female and multimorbidity. The Black and Hispanic respondents differ notably on all covariates except wealth.

### Latent growth models

We specified latent growth models to test our hypotheses and used the Bayesian Information Criterion (BIC) to identify the best fitting models. We examined the intercept of cognition using two measures of discrimination while adjusting for age, female, education, wealth, BMI, physical activity, multimorbidity, neuroticism, and depressive symptoms. We also examined the slope of cognition over time while adjusting for the covariates, but model fit was poorer for most specifications. Adjusting for age in the slope of cognition, however, yielded a better fitting model; therefore, we adjust for age and the respective measures of everyday discrimination. We present parameter estimates for the focal variables in Table 2. Models 1 and 2 of Table 2 examine the relationship between EGD and cognition. Models 3 and 4 examine ERD and cognition. S1 Table provides the unconditional model (A) and full models with all covariates for EGD and ERD (B and C, respectively).

**Table 2:** Latent growth model of alternative everyday discrimination measures predicting cognition.

Variable	Model 1	Model 2	Model 3	Model 4
	Coef (SE)	Coef (SE)	Coef (SE)	Coef (SE)
Intercept				
Constant	17.673***(0.413)	17.650***(0.414)	17.386***(0.409)	17.415***(0.409)
EGD	-0.198***(0.042)	-0.169***(0.048)		
ERD			-0.118(0.074)	-0.335**(0.127)
Black (ref. White)	-2.078***(0.089)	-1.962***(0.120)	-2.046***(0.096)	-2.152***(0.103)
Hispanic	-0.658***(0.112)	-0.624***(0.139)	-0.627***(0.113)	-0.605***(0.118)
*Product terms*				
EGD x Black		-0.140(0.096)		
EGD x Hispanic		-0.050(0.117)		
ERD x Black				0.384*(0.153)
ERD x Hispanic				0.066(0.200)
Slope				
Constant	1.325***(0.105)	1.325***(0.105)	1.378***(0.101)	1.377***(0.101)
EGD	0.034*(0.017)	0.034*(0.017)		
ERD			0.018(0.028)	0.017(0.028)
Likelihood ratio test	χ2(39) = 240.02***	χ2(45) = 243.26***	χ2(39) = 241.93***	χ2(45) = 249.19***
BIC	637,495	641,374	624,055	607,772
N	11,729	11,729	11,729	11,729

Notes: Unstandardized estimates with standard errors in parentheses. BIC = Bayesian information criterion. Models for the intercept adjust for age, female, education, wealth, BMI, physical activity, multimorbidity, neuroticism, and depressive symptoms. Models for the slope adjust for age and the respective type of discrimination. Parameter estimates for the covariates are presented in S1 Table.

*p < .05;

**p < .01;

***p < .001.

As shown in model 1 of Table 2, each additional point of EGD was associated with 0.198-points lower level of cognition at W1 (*p* < 0.001), providing support for hypothesis 1a. Compared to White respondents, Black respondents had initial cognition 2.078-points lower (*p* < 0.001) and Hispanic respondents had initial cognition 0.658-points lower (*p* < 0.001). In the bottom section of Table 2, the slope of EGD was positive (0.034, *p* < 0.05), meaning that people who reported EGD experienced a modest decline compared to those who did not report any EGD. Given that we hypothesized that EGD would be associated with poorer cognition, there is no support for hypothesis 1b.

To test hypothesis 2, we added product terms between EGD and race and ethnicity in model 2 of Table 2, but they were non-significant, meaning that the effect of EGD on initial cognition was not conditional on race or ethnicity. Note also that the BIC in model 2 indicates a poorer model fit than in model 1. We also tested for interactions in the slope of cognition with product terms (EGD x Black; EGD x Hispanic), but the BIC indicated poorer model fit and, therefore, we omitted these variables from the final specification in Table 2. Although we hypothesized that the effect of EGD on initial cognition and change in cognition is moderated by race and ethnicity (poorer cognition), there is no evidence indicative of moderation for hypothesis 2a or 2b.

Models 3 and 4 examined ERD, and the BIC indicated that model 3 was a better fit than either model 1 or 2. From model 3, ERD was not associated with initial cognition (*p* > 0.05), which is inconsistent with hypothesis 3a. Compared to White respondents, Black adults had initial cognition 2.046-points lower, and Hispanic adults had cognition 0.627-points lower (*p* < 0.001). ERD was not associated with the cognition slope in model 3 (no support for hypothesis 3b).

Model 4 is the same as model 3 but with product terms to test for interactions between race and ethnicity and ERD. The BIC for model 4 indicates it is a better fit than model 3. By examining main effects first, the coefficients for ERD and Black are negative (-0.335 [*p* < 0.01] and -2.152 [*p* < 0.001], respectively). The product term, however, reveals that each additional point of ERD for Black respondents resulted in a 0.384 higher value of initial cognition (*p* < 0.05). In other words, although initial cognition was generally lower for Black than White persons, Black people who attributed discrimination to race had better initial cognition scores than Black people who did not report discrimination due to race. This finding provides support for hypothesis 4a. The slope parameters reveal that ERD is not associated with change in cognition over time (*p* > 0.05), and there were no significant interactions between ERD and race and ethnicity on the slope of cognition. There is no evidence to support hypothesis 4b.

### Sensitivity analyses

We conducted several sensitivity analyses. First, given that the relationship between ERD and cognition is conditional on race, we also tested for related three-way interactions (e.g., age, sex) but did not uncover any significant ones. Second, we coded ERD herein for *any* attribution to race but also re-estimated the models with ERD as the *only* reason. Conclusions were very similar, including the interaction of ERD by Black on the intercept of cognition. Third, given that some previous studies include ancestry or national origin along with race in their coding of ERD [10, 33], but others exclude ancestry and national origin from ERD [34, 35], we re-estimated the ERD models presented in Table 2 to examine if adding ancestry or national origin changed the conclusions. We found that the original conclusions were unchanged by replacing ERD with the new variable including ancestry and national origin, and the parameter estimates were very similar. Fourth, we also estimated and present stratified models for White, Black, and Hispanic adults in (S2 and S3 Tables). Most conclusions from the main analysis were similar in the stratified analysis, but we found that the effect of ERD among Black respondents was nonsignificant for the intercept but significant (*p* < .05) and positive for the slope (supporting hypothesis 4b). Fifth, we re-estimated the models on the full sample, before the exclusion criteria were implemented, with full information maximum likelihood models to account for data missingness (i.e., exclusion criteria). Most conclusions were unchanged from Table 2; the only exception was that everyday racial discrimination is significant at *p* < 0.05 in model 3 (see S4 Table). Finally, we also examined additional covariates (e.g., marital status), but these were removed from the final analysis because they were nonsignificant in the models tested.

## Discussion

During the past decade, there have been notable advances as investigators have probed the relationship between discrimination and cognitive health in later life. To our knowledge, this is the first study to systematically compare the prognostic validity of both everyday global discrimination and everyday racial discrimination on cognition over time. The evidence from this study reveals how distinct concepts of discrimination are related to cognition in a diverse sample.

To provide context for our analyses, we begin by noting that the prevalence of discrimination reported in the HRS sample varied notably across the two variables. For the total sample, 65.8% of respondents reported everyday global discrimination, but only 8.3% reported everyday racial discrimination. We expected a difference, but the magnitude of the difference is noteworthy in the HRS sample, which is predominately White and may influence prevalence estimates. Whereas everyday global discrimination measures the gestalt of unfair treatment from all sources, one expects it to be more prevalent than any single attribution. The reported prevalence here for a sample of older adults (mean age = 69) was parallel to two prior studies of adults that compared the wording of discrimination questions in samples with a mean age of 49.6 and 45.1, respectively [9, 10].

In testing our first two hypotheses, we found as expected that everyday global discrimination was associated with poorer initial cognition (hypothesis 1a), which is consistent with prior studies [6, 17]. In hypothesis 1b, we anticipated that everyday global discrimination would be negatively associated with change in cognition but found the opposite: everyday global discrimination was associated with slightly *better* change in cognition over time. Although the effect was modest, the finding is intriguing by showing that the influence of everyday global discrimination was manifest as poorer initial cognition but better cognition several years afterwards. One potential explanation is that sustained cognitive challenges to deal with everyday global discrimination may aid older adults’ cognitive function.

In testing for moderation specified in hypothesis 2, we found that the relationship between everyday global discrimination and cognition was not moderated by race or ethnicity, either for initial cognition or change in cognition. Perhaps this was because much of the reported discrimination associated with the global measure is due to multiple sources.

Related to hypotheses 3a and 3b, our conclusions from the latent growth models showed that everyday racial discrimination was not related to either initial cognition or change in cognition. In testing hypotheses 4a and 4b, we found evidence of moderation by race in the relationship between everyday racial discrimination and initial cognition but not for cognitive change. Initial cognition was generally lower for Black than White persons, but Black people who attributed discrimination to race had better initial cognition scores than those who did not report discrimination due to race. This finding is consistent with results from two prior studies. Sutin and colleagues also analyzed the HRS sample (mean age of 67) and found that Black older adults who reported racial discrimination scored higher on baseline mental status [14]. More recently, Meza and colleagues analyzed cross-sectional data from the Kaiser Healthy Aging and Diverse Life Experiences study reporting that Black older adults (mean age of 76) with higher lifetime racial discrimination had better semantic memory scores than those with no major lifetime discrimination [13]. We are unaware of any studies that show that everyday global discrimination is associated with better initial cognition, but our study adds evidence to the generalization that everyday racial discrimination is related to better initial cognitive functioning among Black older adults.

We offer two potential explanations for why everyday racial discrimination is related to better initial cognitive functioning among Black people. First, we advanced the idea that identifying a specific stressor may be conducive for resilience. Indeed, the act of pinpointing a reason for the discrimination may be more useful than a view that some combination of reasons for the discrimination are involved. Discrimination is rooted in trauma, but stressor specificity may help people to be resilient during the aftermath of unfair treatment [22] and may even evoke distinct neuroendocrine responses [24].

Second, discrimination often sparks a reconsideration of coping strategies and an openness to new strategies. Identifying the discrimination as racial may lead to “collective coping,” which strengthens identity and exposes the person to alternative ways of handling discrimination [23]. Some may think of this as hardiness, but the critical matter is learning, or at least trying alternative coping strategies, to be resilient in the face of discrimination. There also is evidence from the Jackson Heart Study (mean age of 55) that Black people experiencing more everyday discrimination had a slightly lower risk of all-cause mortality [36]. Racial discrimination is morally reprehensible, but those who persisted through such experiences may have adapted coping skills that benefit them in later life [25].

Other explanations are reflected in prior studies reporting that everyday racial discrimination is related to better initial cognitive functioning among Black people. Sutin and colleagues reported parallel findings and speculated that (a) Black people may have support systems that buffer against discrimination, (b) Black people with higher socioeconomic status may simply be more likely to report racial discrimination, and (c) historically disadvantaged groups are more persistent to resolve problems [14]. Meza and colleagues used cross-sectional data and suggested that racial discrimination’s relationship with better cognition in their study may be due to (a) different measures of everyday discrimination, (b) their analysis of major lifetime discrimination, (c) unmeasured confounding, (d) potential reverse causality, and € that social context, especially urban areas, may signal greater prevalence of discrimination and intensify its effect [13].

By studying both initial cognitive function and change in it over time, we found that the direction of the association may vary across the short- or long-term. As such, we believe that future studies comparing the influence of different measures of everyday discrimination on cognitive function over time may help identify how a noxious behavior may have unintended consequences such as helping to preserve cognitive function [26].

It also bears mentioning that initial values of cognition were notably lower for White adults who reported high levels of everyday racial discrimination. White adults’ reports of everyday racial discrimination may pertain to everyday reverse racial discrimination or that one’s spouse or child is non-White, and our results are consistent with another study showing that White people who report unfair treatment due to race have poorer cognition [14]. We are unaware of any evidence for a positive relationship between everyday racial discrimination on cognitive function among White people. Future research on White people reporting everyday racial discrimination is needed to discern why this is the case.

It is also important to recognize the scope and limitations of the findings presented herein. First, we focused on comparing the concepts of everyday global discrimination and everyday racial discrimination, but these analyses could be extended by examining other reasons for unfair treatment (e.g., age, sex). Second, another distinction in the literature on discrimination is between everyday (studied herein) vs. major discrimination (whether the event occurred at any point in the respondent’s life). The HRS measures both but the order of the questions is as follows: (a) everyday discrimination, (b) reason attributed for the everyday unfair treatment, and (c) major discrimination. Attributions are tied explicitly to everyday discrimination, and it is unclear if the attributions also apply to major discrimination. Third, we examined three groups (White, Black, and Hispanic), but other groups such as Native American and Asian also merit attention in future studies. Finally, although the HRS includes oversamples of Black and Hispanic people, the modest sample sizes for these groups is a limitation, especially for selected analyses.

Despite these limitations, these results provide fresh evidence that the relationship between discrimination and cognition varies by the specific concept under investigation and whether one analyzes change in cognition. One implication of this study is to enhance conceptual precision on the topic of discrimination and cognition by not using closely related terms of discrimination interchangeably. Each concept may be wholly appropriate for a given research question, but the interchangeable use of terms that refer to specific discrimination concepts is a problem. In addition, studies that compare results across distinct measures of discrimination may uncover the ways that discrimination is related to health [37].

By comparing two measures of everyday discrimination, our analysis provides compelling evidence that everyday racial discrimination was associated with better initial cognitive function for Black older adults and that everyday global discrimination was associated with better change in cognitive function, regardless of one’s race or ethnicity. Our study helped clarify that discrimination is a multifaceted concept, and specific types of discrimination manifest lower or higher cognitive function during later life for Black, White, and Hispanic adults. These conclusions should not be used to claim that discrimination is beneficial for later life cognition but to stimulate research examining the conditions under which people experiencing discrimination manage to retain their cognitive function despite unfair treatment. From a policy viewpoint, if one seeks to maintain and potentially improve cognition among older adults, studies focused on specific types of discrimination such as race may be more informative than global discrimination for identifying and prioritizing putative targets for intervention.

## Supporting information

S1 Table Complete results from latent growth model of alternative everyday discrimination measures predicting cognition. (PDF)

S2 Table Latent growth model of everyday global discrimination measures predicting cognition stratified by race and ethnicity. (PDF)

S3 Table Latent growth model of everyday racial discrimination measures predicting cognition stratified by race and ethnicity. (PDF)

S4 Table Sensitivity analysis of latent growth model of everyday discrimination measures predicting cognition with full-information maximum likelihood estimation before sample exclusion criteria. (PDF)
