# Supplementary material for: Race, everyday discrimination, and cognitive function in later life
Source: PLoS One. 2023 Oct 25;18(10):e0292617. doi: 10.1371/journal.pone.0292617 (PMC10599523; doi:10.1371/journal.pone.0292617)
Supplement: S1 Table — (PDF) [file pone.0292617.s001.pdf]

SUPPORTING INFORMATION

Race, Everyday Discrimination, and Cognitive Function in Later Life

S1 Table. Complete results from latent growth model of alternative everyday discrimination measures predicting cognition.

	Model A	Model B	Model C
**Variable**	**Intercept**	**Intercept**	**Intercept**
Constant	15.203***(0.038)	17.650***(0.414)	17.415***(0.409)
EGD		-0.169***(0.048)	
ERD			-0.335**(0.127)
Black (ref. White)		-1.962***(0.120)	-2.152***(0.103)
Hispanic		-0.624***(0.139)	-0.605***(0.118)
Age		-0.145***(0.004)	-0.143***(0.004)
Female		0.999***(0.059)	1.019***(0.058)
Education (years)		0.402***(0.011)	0.401***(0.011)
Wealth		0.111***(0.008)	0.112***(0.008)
BMI		0.030***(0.005)	0.029***(0.005)
Physical activity		0.262***(0.064)	0.258***(0.064)
Multimorbidity		-0.212***(0.027)	-0.214***(0.027)
Neuroticism		0.514***(0.080)	0.517***(0.080)
Depressive symptoms		-0.191***(0.016)	-0.201***(0.016)
Product terms			
EGD x Black		-0.140(0.096)	
EGD x Hispanic		-0.050(0.117)	
ERD x Black			0.384*(0.153)
ERD x Hispanic			0.066(0.200)
Slope			
Constant	-0.450***(0.013)	1.325***(0.105)	1.377***(0.101)
EGD		0.034*(0.017)	
ERD			0.017(0.028)
Age		-0.027***(0.001)	-0.027***(0.001)
Likelihood ratio test	$\chi2(5)=149.33$***	$\chi2(45)=243.26$***	$\chi2(45)=249.19$***
BIC	211,377	641,374	607,772
N	11,729	11,729	11,729

Notes: Unstandardized estimates with standard errors in parentheses. Model A is the unconditional model. Models B and C correspond to Models 2 and 4 in Table 2 but display parameter estimates for all covariates.

*p < .05; **p < .01; ***p < .001.
