# Supplementary material for: Race, everyday discrimination, and cognitive function in later life
Source: PLoS One. 2023 Oct 25;18(10):e0292617. doi: 10.1371/journal.pone.0292617 (PMC10599523; doi:10.1371/journal.pone.0292617)
Supplement: S2 Table — (PDF) [file pone.0292617.s002.pdf]

SUPPORTING INFORMATION

Race, Everyday Discrimination, and Cognitive Function in Later Life

S2 Table. Latent growth model of everyday global discrimination measures predicting cognition stratified by race and ethnicity

	White	Black	Hispanic
**Variable**	**Coef (SE)**	**Coef (SE)**	**Coef (SE)**
Intercept			
Constant	17.229***(0.469)	16.968***(1.198)	14.927***(1.386)
EGD	-0.178***(0.049)	-0.311**(0.104)	-0.219(0.137)
Slope			
Constant	1.367***(0.116)	0.846**(0.324)	1.950***(0.394)
EGD	0.031(0.019)	0.054(0.042)	0.053(0.052)
Likelihood ratio test	χ2(33)=164.80***	χ2(33)=92.17***	χ2(33)=57.56***
BIC	496,941	78,420	51,559
N	9,378	1,436	915

Notes: Unstandardized estimates with standard errors in parentheses. BIC = *Bayesian* information criterion. All models adjust for age, female, education, wealth, BMI, physical activity, multimorbidity, neuroticism, and depressive symptoms intercepts and age slope.

*p < .05; **p < .01; ***p < .001.
