# Supplementary material for: Race, everyday discrimination, and cognitive function in later life
Source: PLoS One. 2023 Oct 25;18(10):e0292617. doi: 10.1371/journal.pone.0292617 (PMC10599523; doi:10.1371/journal.pone.0292617)
Supplement: S3 Table — (PDF) [file pone.0292617.s003.pdf]

# SUPPORTING INFORMATION

## Race, Everyday Discrimination, and Cognitive Function in Later Life

**Table S3. Latent growth model of everyday racial discrimination measures predicting cognition stratified by race and ethnicity**

| | **White** | **Black** | **Hispanic** |
|---|---|---|---|
| **Variable** | **Coef (SE)** | **Coef (SE)** | **Coef (SE)** |
| Intercept | | | |
| Constant | 16.997***(0.463) | 16.494***(1.189) | 14.881***(1.380) |
| ERD | -0.252(0.136) | -0.051(0.108) | -0.365(0.193) |
| | | | |
| Slope | | | |
| Constant | 1.424***(0.111) | 0.787*(0.319) | 1.959***(0.387) |
| ERD | -0.064(0.057) | 0.101*(0.043) | 0.107(0.074) |
| Likelihood ratio test | $\chi$2(33)=162.44*** | $\chi$2(33)=91.97*** | $\chi$2(33)=57.21*** |
| BIC | 477,707 | 78,347 | 50,943 |
| N | 9,378 | 1,436 | 915 |

Notes: Unstandardized estimates with standard errors in parentheses. BIC = *Bayesian* information criterion. All models adjust for age, female, education, wealth, BMI, physical activity, multimorbidity, neuroticism, and depressive symptoms intercepts and age slope.

*p < .05; **p < .01; ***p < .001.
