# Supplementary material for: Race, everyday discrimination, and cognitive function in later life
Source: PLoS One. 2023 Oct 25;18(10):e0292617. doi: 10.1371/journal.pone.0292617 (PMC10599523; doi:10.1371/journal.pone.0292617)
Supplement: S4 Table — (PDF) [file pone.0292617.s004.pdf]

SUPPORTING INFORMATION

Race, Everyday Discrimination, and Cognitive Function in Later Life

S4 Table. **Sensitivity analysis of latent growth model of everyday discrimination measures predicting cognition with full-information maximum likelihood estimation before sample exclusion criteria**

	Model 1	Model 2	Model 3	Model 4
**Variable**	**Coef (SE)**	**Coef (SE)**	**Coef (SE)**	**Coef (SE)**
Intercept				
Constant	16.183***(0.368)	16.178***(0.368)	15.879***(0.363)	15.953***(0.363)
EGD	-0.211***(0.040)	-0.205***(0.045)		
ERD			-0.139*(0.066)	-0.476***(0.102)
Black (ref. White)	-2.175***(0.079)	-2.142***(0.111)	-2.123***(0.085)	-2.312***(0.094)
Hispanic	-0.629***(0.098)	-0.630***(0.125)	-0.585***(0.098)	-0.591***(0.104)
Product terms				
EGD x Black		-0.038(0.091)		
EGD x Hispanic		0.001(0.111)		
ERD x Black				0.631***(0.131)
ERD x Hispanic				0.260(0.170)
Slope				
Constant	1.053***(0.084)	1.053***(0.084)	1.107***(0.080)	1.107***(0.080)
EGD	0.030*(0.015)	0.030*(0.015)		
ERD			-0.001(0.024)	-0.002(0.024)
Likelihood ratio test	$\chi2(39)$=328.12***	$\chi2(45)$=331.00***	$\chi2(39)$=329.28***	$\chi2(45)$=335.62***
BIC	894,189	901,308	879,173	865,552
N	20,753	20,753	20,753	20,753

Notes: Unstandardized estimates with standard errors in parentheses. BIC = *Bayesian* information criterion. Product terms for discrimination correspond to the type of discrimination (EGD or ERD) in each model. Models for the intercept adjust for age, female, education, wealth, BMI, physical activity, multimorbidity, neuroticism, and depressive symptoms. Models for the slope adjust for age and the respective type of discrimination.

*p < .05; **p < .01; ***p < .001.
